# Redox protection by bacterial glutathione peroxidase drives virulence in *Pseudomonas aeruginosa*

**DOI:** 10.1080/13510002.2026.2676357

**Published:** 2026-05-22

**Authors:** Jadye da Silva Cordeiro, Thiago Macêdo Lopes Correia, Igor Pereira Ribeiro Muniz, Railmara Pereira da Silva, Talita Costa dos Santos, Leonardo Silva Rocha, Regiane Yatsuda, Julia de Oliveira Borges, Albert Souza Peixoto, Amélia Cristina Mendes de Magalhães, Robson Amaro Augusto da Silva, Flávia Carla Meotti, Raphael Ferreira Queiroz

**Affiliations:** a Programa Multicêntrico de Pós-Graduação em Bioquímica e Biologia Molecular, Universidade Estadual do Sudoeste da Bahia, Vitória da Conquista, Brazil; b Programa Multicêntrico de Pós-Graduação em Ciências Fisiológicas, Universidade Federal da Bahia, Campus Anísio Teixeira, Vitória da Conquista, Brazil; c Departamento de Bioquímica, Instituto de Química, Universidade de São Paulo, Sao Paulo, Brazil; d Departamento de Fisiologia e Biofísica, Instituto de Ciências Biomédicas, Universidade de São Paulo, Sao Paulo, Brazil; e Departamento de Ciências da Saúde, Universidade Estadual do Sudoeste da Bahia, Vitória da Conquista, Brazil

**Keywords:** Glutathione peroxidase, *Pseudomonas aeruginosa*, redox stress, hyperuricemia, virulence factors, redox regulation, antioxidant enzymes, host–pathogen interaction

## Abstract

**Background:**

*Pseudomonas aeruginosa* relies on antioxidant enzymes to withstand host-derived oxidative stress during infection. Here, we investigated the role of bacterial glutathione peroxidase (GPx) in survival and virulence of the PA14 strain under inflammatory conditions associated with hyperuricemia.

**Methods:**

Wild-type (WT) and *Δgpx* strains were exposed to isolated hypochlorous acid (HOCl), hydrogen peroxide (H₂O₂), tert-butyl hydroperoxide, and urate hydroperoxide (HOOU) or challenged with human neutrophils in the presence or absence of oxidative burst modulators. *In vivo*, normouricemic and potassium oxonate–induced hyperuricemic mice were intranasally infected with WT or *Δgpx* strains to assess bacterial burden, inflammation, oxidative damage, and survival.

**Results:**

Deletion of *gpx* increased bacterial susceptibility to all oxidants, particularly HOOU, and significantly reduced survival in activated neutrophils. In mice, *Δgpx* infection resulted in reduced pulmonary bacterial burden, attenuated neutrophil infiltration, lower oxidative damage, and markedly improved survival compared to WT infection, an effect exacerbated by hyperuricemia in WT-infected animals. These findings demonstrate that GPx detoxifies organic hydroperoxides generated during inflammation, enhancing *P. aeruginosa* resistance to oxidative killing and promoting virulence.

**Conclusion:**

Collectively, our results highlight the importance of redox regulation in bacterial pathogenesis and identify GPx as a potential target for redox-based anti-virulence strategies against multidrug-resistant *P. aeruginosa*.

## Introduction

*Pseudomonas aeruginosa* is a gram-negative opportunistic bacterium that causes severe infections within diverse host tissues [1]. Its pathogenicity is enhanced by the ability to form biofilms, resistance mechanisms, and virulence determinants, including type III secreted proteins, quorum-sensing systems, elastases, alginate, and siderophores [2]. These attributes allow *P. aeruginosa* to persist in hostile host environments and evade immune clearance. *Pseudomonas aeruginosa* frequently causes nosocomial infections that predominantly affect immunocompromised patients (e.g. cystic fibrosis or burn injuries) and are associated with high rates of mortality and morbidity [3,4].

The ESKAPE group, comprising the *Enterococcus faecium, Staphylococcus aureus, Klebsiella pneumoniae, Acinetobacter baumannii, Pseudomonas aeruginosa,* and *Enterobacter* species, represents a cluster of highly antimicrobial-resistant pathogens of major clinical relevance. *P. aeruginosa* is a prominent member of this group that poses a significant threat to global public health and is responsible for nearly 20% of all antibiotic resistance–related deaths [5–7]. Its remarkable metabolic versatility and ability to rapidly adapt to hostile environments make treatment a continuous challenge, highlighting the urgent need for novel therapeutic approaches beyond classical antibiotics [8].

*Pseudomonas aeruginosa* is a clinically and hospital-relevant pathogen with a prevalence rate between 7.1% and 7.3% that is frequently identified in infections related to pneumonia, surgical procedures, urinary tract infections, and bacteremia [5–7,9,10]. During infection, intense recruitment and activation of neutrophils constitute a central component of the host's innate immune response, particularly in the lungs. Activated neutrophils generate large amounts of reactive oxygen species and release extracellular traps to combat the pathogen [11,12]. The oxidative burst, characterized by the production of oxidants, such as HOCl and H₂O₂, represents a key antimicrobial mechanism for eliminating *P. aeruginosa* [13,14]. This oxidative pressure imposes a substantial redox challenge on the bacterium, necessitating robust antioxidant defenses to maintain intracellular redox homeostasis [15,16]. Among these, glutathione peroxidases (GPx) reduce organic hydroperoxides using reduced glutathione as an electron donor. However, the role of GPx seems to differ across bacterial species, reflecting functional diversity [17,18]. For example, Zhang et al. [19] observed that the absence of the GPx enzyme in *Listeria monocytogenes* led to increased bacterial resistance to redox stress caused by metal ions, whereas our group previously demonstrated that PA14 carrying a *gpx* mutation exhibited increased sensitivity to HOCl compared with the wild-type (WT) strain [15].

Uric acid (UA), a well-known antioxidant present in physiological fluids at concentrations ranging from 50 to 400 μM [20,21], is susceptible to oxidation by the peroxidase cycle of MPO *in vivo*. This reaction converts UA to urate free radical, which adds to superoxide forming urate hydroperoxide (HOOU), a moderate oxidant that reacts with thiol-peroxidases and other thiol proteins [22–25]. Upon oxidation by MPO, UA diverts the chlorinating cycle of the enzyme and leads to decreased HOCl production and impaired bactericidal activity of MPO against PA14 (i.e. a highly virulent *P. aeruginosa* strain) [23]. Concurrently, HOOU is generated and subsequently neutralized by bacterial peroxidases [15]. Thus, UA exerts a dual role during infection, simultaneously dampening HOCl-mediated bacterial killing while generating secondary oxidants that challenge bacterial redox homeostasis. In addition, elevated concentrations promote monosodium urate crystal formation and NLRP3 inflammasome activation [23,26], and under certain oxidative conditions, it may also display pro-oxidant behavior [21].

This study investigates the role of GPx from *P. aeruginosa* PA14 in bacterial resistance to oxidative stress and virulence during acute pulmonary infection under hyperuricemic conditions. Specifically, we sought to determine whether GPx contributes to bacterial fitness during oxidative burst–driven inflammation. We showed that deletion of GPx increases bacterial susceptibility to multiple oxidants and to neutrophil-mediated killing. While infection with the WT strain resulted in rapid mortality, particularly in hyperuricemic mice, animals infected with the *Δgpx* mutant survived despite the pro-inflammatory environment. These findings indicate that GPx is a key determinant of *P. aeruginosa* fitness during oxidative burst–driven inflammation and is required to sustain pulmonary infection and disease severity *in vivo*.

## Material and methods

### Materials

The following formulations and reagents were purchased from Sigma-Aldrich (Sigma-Aldrich, MA, U.S.A.): sodium chloride (S9888), calcium chloride (C1016), magnesium chloride (M8266), sodium phosphate monobasic (S9638), sodium phosphate dibasic (S0876), potassium phosphate monobasic (P0662), sodium hydroxide (S5881), potassium chloride (P4504), ammonium sulfate (A4418), diethylenetriaminepentaacetic acid (D6518), ammonium acetate (A1542), acetonitrile (34998), riboflavin (R9504), Chelex-100 (C7901), Luria-Bertani (LB) broth (L3397), Müeller Hinton (MH) agar (70191), ampicillin (A9518), Triton X-100 (T9284), 3,3′,5,5′-tetramethylbenzidine (TMB) (T0440-100:mL), H₂O₂ (216763), 4-aminobenzoic acid hydrazide (ABAH) (A4297), apocynin (A1146), tert-butyl Hydroperoxide (458139), sodium carboxymethylcellulose (medium viscosity, C4888-500G), oxonic acid potassium salt (156124), cetyltrimethylammonium bromide (H9151), trypan blue (T854), dimethyl sulfoxide (D2650), naphthylethylenediamine (222488), sulfanilamide (S9251), bradford (B6916-500 mL), bovine serum albumin (A2153-10G), glycerol (G5516), anhydrous glucose (G5767), Histopaque-1077, Histopaque-1119, HOCl (42044), 3,3′-diaminobenzidine (750118), methylene blue (M9140), acetic acid (A6283), hydrated ethyl alcohol (459836), sodium azide (S2002-5G), antibody against protein carbonyl (anti-DNP) (D9656), Harris hematoxylin (HHS32), butylated hydroxytoluene (PHR1117-1G), Tris(hydroxymethyl)aminomethane (TA09508BRA), hydrochloric acid (AC09087SO), horseradish peroxidase (P6782), and cytochalasin B (C6762). Ketamine and Xylazine were purchased from Syntec, Barueri, São Paulo, Brazil. UA (K139-1) was purchased from Bioclin, Minas Gerais, Brazil. Eosin (EA36) and Hematoxylin (620503) were purchased from Laborclin, Pinhais, Paraná, Brazil. Avidin-biotin-peroxidase complex was purchased from Vector Laboratories, California, U.S.A. IL-1β (DY201-05) and TNF-α (DY410-05) cytokines were purchased R&D Systems Minneapolis, Minnesota, U.S.A. Xylene (X09738RA) and paraffin (PH06788BRA) were purchased from Êxodo Científica, Hortolândia, São Paulo, Brazil. Methanol (A1085.01.BJ), chloroform (C1062.01.BJ), tween 20 (T1028.02.BJ), trichloroacetic acid (A1066.01.AG) were purchased from Synth, Diadema, São Paulo, Brazil. Platinum™ Taq DNA Polymerase (10966018) was acquired from Invitrogen, Carlsbad, California, U.S.A. HOCl stock solutions were freshly prepared, and concentrations were determined spectrophotometrically in NaOH (0.01 M) (λ_290_ nm = 3.5 × 10² M^−1^·cm^−1^) [27]. Fresh stock solutions of H_2_O_2_ were prepared before use, and concentrations were determined spectrophotometrically by reacting with horseradish peroxidase to form compound I (λ_403_ nm = 5.4 × 10⁴ M^−1^·cm^−1^) [28]. The minimal medium was prepared using sodium phosphate monobasic (31 mM), sodium phosphate dibasic (39 mM), ammonium sulfate (18 mM), calcium chloride (100 μM), magnesium chloride (2 mM), diethylenetriaminepentaacetic acid (100 μM), and anhydrous glucose (2%) at pH 7.4. Phosphate-buffered saline with glucose (PBS–glucose) was prepared using sodium phosphate dibasic (10 mM), potassium phosphate monobasic (1.8 mM), sodium chloride (137 mM), potassium chloride (2.7 mM), and anhydrous glucose (1%) at pH 7.4. All solutions were prepared with sterile ultrapure water (Hidrotek RO7).

### Bacterial strains

*P. aeruginosa* UCBPP-PA14 (PA14) wild-type and the Δ*gpx* mutant (PA14_27520) obtained from the Harvard transposon insertion mutant library of *P. aeruginosa* strain PA14, were kindly provided by Prof. Dr. Regina Baldini, Instituto de Química, Universidade de São Paulo, São Paulo, Brazil [29,30]. All experiments involving PA14 were conducted in certified biosafety level 2 (BSL-2) facilities in accordance with institutional biosafety regulations and approved by the Institutional Biosafety Committee (SEI number: 01245.007935/2021-40).

### PCR confirmation of *gpx* deletion

To confirm deletion of the *gpx* gene in the *Δgpx* strain, colony PCR was performed on individual colonies of *P. aeruginosa* PA14 WT, *Δgpx* mutant and complemented (*Δgpx attB:gpx*) strains. Amplification of the gpx locus (PA14_27520) was carried out using forward primer 5’-GAACTACGGGGTGAGCTTCC-3’ and reverse primer 5’-AGTTCCACTTGATGCCCTGG-3’, generating a 128 bp product. As an internal control, the constitutively expressed housekeeping gene *nadB* [31] was amplified using forward primer 5’-CTACCTGGACATCAGCCACA-3’ and reverse primer 5’-GGTAATGTCGATGCCGAAGT-3’, yielding a 94 bp amplicon to confirm template integrity and amplification efficiency. Primer specificity was confirmed by NCBI.

Primer-BLAST against the *P*. aeruginosa PA14 genome (GenBank accession no. CP000438.1), predicting single amplicons mapping to genomic coordinates 2389194–2389321 (PA14_27520, *gpx*) and 4827988–4828081 (PA14_54450, *nadB*). PCR reactions were prepared using Taq DNA Polymerase in the supplied reaction buffer, containing 250  μM dNTPs and 0.5  μM of each primer. Thermal cycling conditions consisted of an initial denaturation step at 94 °C for 3 min, followed by 40 cycles of denaturation at 94 °C for 30 s, annealing at 60 °C for 1 min, and extension at 72 °C for 1 min, with a final extension at 72 °C for 2 min. PCR products were resolved by agarose gel electrophoresis and visualized under UV illumination.

### Determination of the bacterial growth curve

The Δ*gpx* and WT strains were cultured in LB broth at 37 °C for 18 h under agitation (350 rpm) in a bacteriological incubator shaker (Loccus Biotecnologia, São Paulo, Brazil). Subsequently, the WT and mutant strains were diluted to an OD_625 nm_ of 0.1 in LB broth and incubated at 37 °C under agitation (350 rpm). At 0, 1, 2, 3, 4, 5, and 6 h, the absorbance of each tube was measured at OD_625_ nm. These suspensions were also diluted 10⁴-fold in minimal medium without glucose, and 10 μL aliquots were plated on MH agar. After incubation for 18 h at 30 °C, the colonies were counted to determine the number of colony-forming units (CFU)/mL [15,23].

### Chemical preparation and quantification of HOOU

HOOU was synthesized according to [32,33]. Briefly, urate solutions (20 mM) were prepared in NaOH (40 mM), and riboflavin (500 μM) was dissolved in phosphate buffer (20 mM; pH 6.0). Urate (1.5 mM) and riboflavin (0.01 mM) were mixed in 6-well plates in a final volume of 4 mL of phosphate buffer (20 mM, pH 6.8). The mixture was stirred with Chelex resin for at least 1 h to remove trace metals. Subsequently, the plates were irradiated with UV-A light (365 nm, UV-A irradiator with six lamps, 15 and 2.2 mW/cm²; Novatecnica, Campinas, Brazil) for 10 min. The reaction mixture was injected into a high-performance liquid chromatography system (Shimadzu, Tokyo, Japan) equipped with two LC-6AD pumps, one CTO-10A manual injector, one SPD-20A UV absorbance detector, and one CBM-20A system controller connected to a computer with the LC Software Solution. A preparative TSK-Gel amide-80 column (21.5 mm × 30 cm, 10 μm particle size; Tosoh Bioscience, Tokyo, Japan) was used as the stationary phase. The mobile phase consisted of ammonium acetate (10 mM, pH 6.8; solvent A) and acetonitrile (solvent B). Before injection, the reaction mixture was diluted (40% reaction mixture and 60% acetonitrile), and 7 mL was injected into the high-performance liquid chromatography system. The sample was separated under isocratic conditions using a 60% solvent B and 40% solvent A mixture for 30 min at a flow rate of 4.0 mL/min. Approximately 4 mL of HOOU (retention time of 18 min) was collected and exposed to argon to evaporate the acetonitrile. Residual acetonitrile was removed using a Kitasato flask connected to a vacuum pump for 5 min. The HOOU concentration was determined spectrophotometrically at 308 nm (*ε* = 6537 M⁻¹·cm⁻¹) [33] and confirmed by the FOX method using H₂O₂ as a standard [34].

### Evaluation of bactericidal activity by HOCl, H_2_O_2_, *t*BOOH, and HOOU

The Δ*gpx* and WT strains were also grown in LB broth at 37 °C for 18 h under agitation (350 rpm) in the bacteriological incubator shaker. Subsequently, cells were diluted to an OD_625 nm_ of 0.1 in LB broth and incubated again for 5 h. After incubation, the cultures were diluted in minimal medium to a concentration of 1.0 × 10^6^ CFU/mL and treated with HOCl, H_2_O_2_, *t-*BOOH or HOOU (0 to 40 µM, 50 to 2000 µM, and 25 to 1000 µM, 0 to 80 µM, respectively) for 30 min  at 37 °C. The reaction mixture was diluted 200-fold in LB broth to neutralize residual peroxides, and 10 μL aliquots were streaked onto MH agar plates, which were incubated at 30 °C for 18 h. Bactericidal activity was estimated by counting the number of residual CFU [15,23].

### Isolation of neutrophils from human peripheral blood

Blood samples from healthy volunteers of both sexes (age 20 to 40 years and non-smokers) were obtained via peripheral venipuncture and collected into heparinized tubes. Granulocyte isolation was performed according to [35] with modifications. A total of 3 mL of Histopaque-1119 was added to a sterile 15 mL conical tube at room temperature, followed by 2 mL of Histopaque-1077 and 4 mL of heparinized blood. The material was centrifuged at 700 × *g* (Fanem 206-LB, São Paulo, Brazil) for 30 min at room temperature. After centrifugation, the supernatant was discarded, and the granulocyte-rich interface between the two gradients was aspirated using a Pasteur pipette. The cells were washed with 10 mL of sterile saline solution (0.9%) and centrifuged at 300 ×* g* for 10 min. Following supernatant removal, the pellet underwent hypotonic lysis with 5 mL of ice-cold distilled water to remove the remaining erythrocytes. The suspension was centrifuged again at 300 ×​​​​​ *g* for 10 min, the supernatant was discarded, and the pellet was resuspended in 2 mL of PBS-glucose buffer. Cell counting was performed using a Neubauer chamber (Sigma-Aldrich, MA, U.S.A), while cell viability was assessed using the trypan blue (0.2%) exclusion assay [36]. The study was approved by the Human Research Ethics Committee of the Universidade Estadual do Sudoeste da Bahia (protocol number CAAE 46135315.4.0000.0055) and conducted in accordance with the Declaration of Helsinki. Written informed consent was obtained from all participants.

### Evaluation of neutrophil bactericidal activity

After bacterial growth in a bacteriological incubator for 18 h at 37 °C and agitation at 350 rpm, *Δgpx,* and WT were diluted in LB broth to OD625 nm = 0.1 and incubated again for 5 h. The cells were centrifuged at 1700 × *g* (Fanem 3400, São Paulo, Brazil) for 10 min and resuspended in PBS-glucose buffer. Subsequently, 10% heat-inactivated human serum was added, and the suspension was incubated at 37 °C for 20 min to allow opsonization. Neutrophils (1.0 × 10^6^ cells/mL) were challenged with bacteria (1.0 × 10^7^ CFU/mL) in PBS-glucose at a multiplicity of infection of 10:1, in the absence or presence of the following compounds: UA (100, 200, and 400 µM); HOOU (0 to 80 µM); ABAH (50 µM), which is an MPO inhibitor [37,38]; apocynin (1 mM), a NADPH oxidase inhibitor [39,40]; or cytochalasin B (10 µg/mL), a phagocytosis inhibitor [41]. Stock solutions of ABAH, apocynin, and cytochalasin B were prepared in dimethyl sulfoxide (DMSO). HOOU was prepared as described above, and UA was dissolved in an alkaline solution followed by pH neutralization. Vehicle controls contained DMSO at a final concentration of 0.001%, which had no effect on neutrophil or bacterial viability. Triton X-100 (10%) was added to the reaction mixture after 1 h of incubation to induce complete neutrophil lysis. After 10 min on ice, the homogenates were diluted 10^4^-fold, and 10 µL were plated onto MH agar plates and incubated at 30 °C for 18 h. Bactericidal activity was assessed by counting the residual CFU [15,23].

### Animal model

Forty-eight male C57BL/6 mice aged eight weeks were obtained from the Anilab (Paulínia, São Paulo, Brazil) and housed for acclimatization for 10 days in the animal facility of the Instituto Multidisciplinar de Saúde of the Universidade Federal da Bahia. The animals were maintained under a 12-h light/dark cycle in pathogen-free conditions with controlled temperature (23 °C ± 3 °C) and *ad libitum* access to standard commercial chow and water (Pragsoluções, São Paulo, Brazil). All procedures were conducted according to the National Council for the Control of Animal Experimentation and the Ethical Principles in Animal Experimentation, and were approved by the research ethics committee on animal use of the Universidade Estadual do Sudoeste da Bahia (number 171/2023).

For each experimental group, a total of eight animals were used. Due to logistical constraints, infections were performed in two separate experimental batches, each including half of the animals per group. All procedures, inoculum preparation, infection conditions, and experimental endpoints were identical between batches. Animals from both batches were considered biological replicates within a single experimental design and pooled for statistical analysis.

### Induction of hyperuricemia

Mice received daily intraperitoneal injections of potassium oxonate (300 mg/kg) to induce hyperuricemia [42]. Sodium carboxymethylcellulose (0.5%) was dissolved in sterile saline, and potassium oxonate was freshly dissolved daily in 0.5% sodium carboxymethylcellulose solution before intraperitoneal administration. The treatment started one day before bacterial infection and continued once daily throughout the experimental period until completion of 3 days (tissue collection) and 17 days (survival curve).

### Intranasal infection

After culturing the Δ*gpx* and WT PA14 strains, the bacteria were diluted to OD₆₂₅ _nm_ = 0.1 and centrifuged at 4500 × *g* for 8 min, and the pellet was resuspended to a concentration of 3.0 × 10^6^ cells in 30 µL of sterile 0.9% saline (6.7 × 10^7^ bacteria/mL). The animals were anesthetized with ketamine (50 mg/kg, i.p.) and xylazine (16 mg/kg, i.p.) [43]. Normouricemic and hyperuricemic animals (*n* = 8 for each bacterial strain) were infected intranasally with 30 µL of the bacterial suspension [15,43–46]. The control group (*n* = 8) was inoculated intranasally with 30 μL of sterile saline.

### Survival curve

To minimize suffering, the animals were euthanized by cervical dislocation upon reaching a moribund state based on the following criteria: inability to remain upright (with or without respiratory distress and cyanosis), anorexia and weight loss (>20%), body curvature, prostration, impaired mobility, unkempt fur, and signs of dehydration [47]. The animals were observed twice daily for survival up to 15 days post-bacterial instillation. On the 16^th^ day, the animals were anesthetized intraperitoneally with a mixture of xylazine (16 mg/kg) and ketamine (50 mg/kg) and decapitated to determine serum UA and cytokines. Blood samples were centrifuged at 1500 × *g* for 10 min at room temperature, and serum was collected and stored at −80 °C for UA and cytokine measurements.

### Uric acid determination

Serum UA was assessed using a monoreagent kit (Bioclin, Minas Gerais, Brazil) according to the specifications of the manufacturer.

### Bronchoalveolar lavage and organ collection

After euthanasia, mice underwent tracheal cannulation using a G22 infusion catheter, and broncoalveolar lavage (BAL) was obtained by intratracheal instillation and recovery of a total volume of 1.5 mL of PBS-glucose (three washes of 500 µL each). Samples were kept on ice throughout the procedure to prevent cell lysis. The lungs, liver, and spleen were collected by median laparotomy and stored according to the requirements of each subsequent analysis. Carcasses were stored at –20 °C for incineration.

### Total and differential leukocyte count in BAL

For total leukocyte counting, an aliquot of BAL was diluted in Turk's solution to lyse residual erythrocytes prior to total cell counting using a Neubauer chamber (NewProv, Paraná, Brazil). For differential leukocyte analysis, 50 µL of BAL was cytocentrifuged at 4500 × *g* (Presvac, Santa Catarina, Brazil) for 7 min. The slides were stained with the Rapid Panoptic kit (NewProv, Paraná, Brazil) by immersion in three different solutions: methanol, eosin, and methylene blue. Based on standard morphological criteria [48,49], two independent analysts randomly counted 100 cells per slide to differentiate the leukocytes. The remaining BAL was centrifuged at 1500 × *g* for 10 min at 4 °C (Z 36 HK, Hermle-Labortechnik, Germany), and the supernatant was stored at –80 °C for analysis.

### Determination of nitrite levels in BAL

Nitrite levels in BAL supernatant were measured using the Griess method [50]. The sample (50 μL) was incubated with 50 μL of naphthylethylenediamine solution (0.1%) and 50 μL of sulfanilamide solution (1%) for 10 min at 25 °C. The absorbance of the samples was measured at 540 nm and interpolated on a nitrite standard curve (1.65 to 100 μM).

### Colony-forming units in tissues

The organs (~100 mg of the right lung, liver, and spleen) were immediately homogenized in PBS-glucose buffer at a concentration of 100 mg/mL using the TissueRuptor system (Qiagen, Maryland, U.S.A.). A 10 μL aliquot of each organ homogenate was serially diluted up to 10^4^-fold and plated on MH agar. After 18 h at 30 °C, residual colonies were counted under a 10²-fold dilution, and colony-forming units were expressed as CFU/g of tissue [15,51].

### Protein concentration in samples

Protein concentration in lung tissue homogenates was determined using the Bradford colorimetric method, with absorbance measured at 595 nm [52]. Bovine serum albumin (Sigma-Aldrich) (0 to 1.4 mg/mL) was used as a standard.

### Peroxidase activity in the lungs

Peroxidase activity was measured using TMB [53–55]. The lung tissue was homogenized in 50 mM potassium phosphate buffer, pH 6.0, at a concentration of 100 mg/mL, and centrifuged (10,000 × *g*) for 10 min at 4 °C. Cetyltrimethylammonium bromide 0.5% was added to the supernate. Subsequently, 110 µL of TMB solution (2.9 mM in dimethyl sulfoxide 14% and 150 mM sodium phosphate buffer, pH 5.4) was added to the homogenate, followed by 80 µL H_2_O_2_ (0.75 mM) to trigger the reaction. TMB oxidation was measured every 30 s at room temperature for 5 min at 450 nm (ϵ_450 nm_ = 5.9 × 10⁴ M^−1^∙cm^−1^). As a control, ABAH (100 µM) was incubated with the homogenate from WT-infected mice to assess the involvement of MPO in TMB oxidation [37,38]. Results were expressed as specific activity (U/mg protein), in which one unit of peroxidase activity was defined as the amount of enzyme that produced 1 μmol of oxidized TMB per minute.

### Histological analyses

The left lung was removed, fixed in methacarn solution (60% methanol, 30% chloroform, and 10% acetic acid) for 24 h, and transferred to 70% hydrated ethyl alcohol. Subsequently, the tissue was dehydrated in a graded alcohol series, cleared in xylene, paraffin-embedded, sectioned into 4 μm-thick slices using a microtome (Leica RM2125RTS, China), and stained with hematoxylin and eosin for histological analysis. A total of 20 fields of view at 200× magnification were selected and assessed by two trained evaluators using ImageJ software, version 1.54 g.

### Immunohistochemical analysis

The histological sections were subjected to deparaffinization and hydration, followed by antigen retrieval performed with phosphate buffer [55,56]. Endogenous peroxidase inhibition was achieved by incubating the sections with sodium azide (0.1%) and H_2_O_2_ (0.3%) for 10 min. The slides were washed twice for 5 min with PBST (PBS containing 0.1% Tween 20), and the nonspecific binding of immunoglobulins was blocked with a 20% goat serum solution. After 30 min, the sections were incubated overnight in the refrigerator with the primary antibody against protein carbonyl (anti-DNP) (dilution 1:100). Subsequently, the slides were washed twice with PBST for 5 min, followed by incubation with the secondary antibody for 30 min at room temperature. The reaction product was detected by incubating the sections with avidin-biotin-peroxidase complex for 30 min, and the color was developed after approximately 2 min of stable DAB addition. The slides were counterstained using Harris hematoxylin. Ten random fields were photographed using a digital camera (4K HDMI 48MP, New Value) attached to a light microscope (P 207 T LED) at 400x magnification. The immunostaining for DNP was expressed as the percentage of the marked area/µm² relative to the total area. The analysis was performed using ImageJ software, version 1.54 g.

### Quantification of thiobarbituric acid reactive products

The lipid peroxidation was determined by TBARTs method according to [57]. The right lung tissue (100 mg/mL) was homogenized in 50 mM Tris–HCl buffer, pH 8, with the presence of butylated hydroxytoluene (0.02%) to prevent spurious oxidation. The homogenate was centrifuged at 1600 × *g* for 10 min at 4 °C. The supernatant was collected, diluted (1:2) in 100 μL of Tris(hydroxymethyl)aminomethane (TEP) adjusted with hydrochloric acid (Tris–HCl), and incubated with 100 μL of trichloroacetic acid (10%) and 800 μL of thiobarbituric acid (0.53% dissolved in 20% acetic acid) for 1 h at 95 °C. After incubation in a water bath, samples were iced for 10 min and centrifuged again at 1600 × *g* for 10 min at 4 °C. Absorbance was measured at 535 nm, and the levels of thiobarbituric acid reactive products were quantified by interpolating sample absorbance against a standard TEP curve (0 to 50 μM). Results were normalized by protein concentration and expressed as μmol of TEP per gram of protein.

### Cytokines levels

IL-1β and TNF-α cytokines in serum or lung homogenates were determined using enzyme-linked immunosorbent assay kits according to the manufacturer’s instructions (R&D Systems), except for the first step, incubation of samples for 18 h at room temperature. Cytokine concentrations were expressed in pg/mL (serum) or pg/mg of protein (lung homogenates).

### Statistical analysis

Data are presented as mean ± standard error of the mean, with the number of replicates indicated in the figure legends. Data normality was assessed using the Shapiro–Wilk test, and comparisons were performed using the one-way ANOVA (Bonferroni or Newman–Keuls post-hoc) or comparisons between two groups were performed using the unpaired Student's *t*-test. Significance was set at *p* < 0.05. All analyses were performed using the GraphPad Prism software (La Jolla, California, U.S.A.) version 5.0.

## Results

Deletion of the *gpx* gene in the *Δgpx* strain was confirmed by PCR using gene-specific primers. Amplification of *gpx* was detected in the wild-type strain and *Δgpx attB:gpx*, but not in the *Δgpx* strain, while a constitutively expressed gene was amplified in all strains, confirming DNA integrity (Supplementary Figure S1).

Next, the growth of WT PA14 and *Δgpx* strains was assessed by monitoring optical density at 625 nm and bacterial counts (CFU/mL) over a 6-h period (Supplementary Figure S2A and S2B, respectively). Growth curves obtained by both methods were fully overlapping, indicating that deletion of gpx does not affect bacterial growth kinetics in broth compared with the WT strain. Importantly, CFU-based enumeration independently validated the OD₆₂₅ measurements, confirming equivalent growth of WT and *Δgpx* strains.

**Figure 1.**
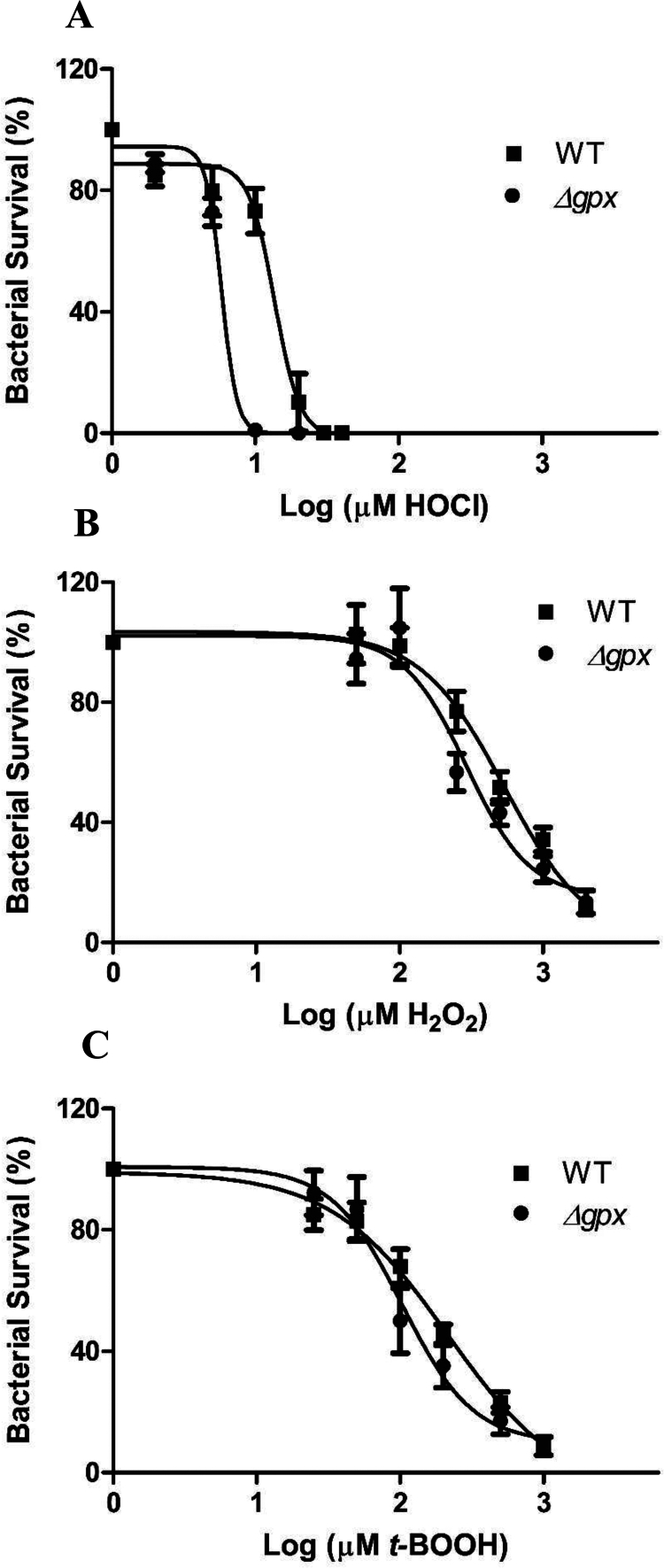
Growth inhibition of the wild-type (WT) and *∆gpx* PA14 strains by hypochlorous acid (HOCl) (A), hydrogen peroxide (H2O2) (B), and tert-butyl hydroperoxide (*t*BOOH) (C). Data presented as mean ± standard error of triplicates from five independent experiments. Dose–response curves were fitted to a sigmoidal model for *∆gpx* and WT strains using GraphPad Prism 5.0 software.

Compared with the WT strain, the *∆gpx* was more sensitive to HOCl (IC50 = 13.61 ± 0.04 vs. 5.81 ± 0.02, *p* < 0.0001) (Figure 1A), H2O2 (IC50 = 541.8 ± 0.1 vs. 293.4 ± 0.1, *p* < 0.0001) (Figure 1B), and *t-*BOOH (IC50 = 215.4 ± 0.1 vs. 103.4 ± 0.1, *p* < 0.0001) (Figure 1C).

**Figure 2.**
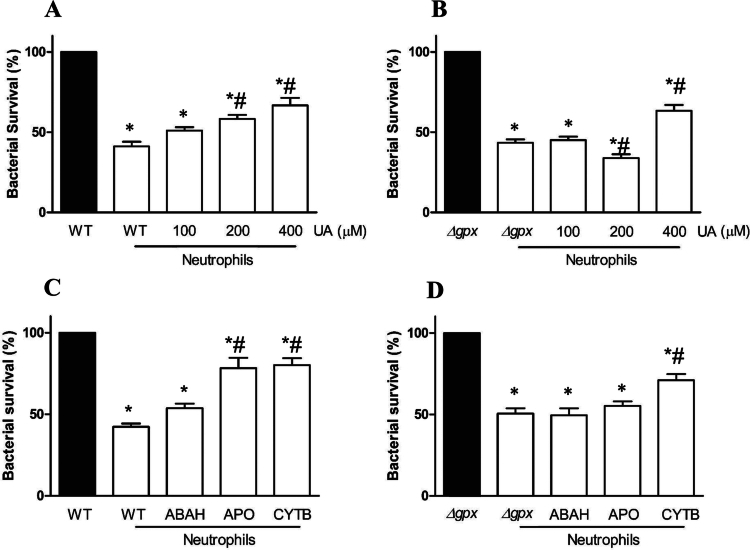
Survival of the wild-type (WT) and ∆*gpx* PA14 strains following neutrophil oxidative burst. WT and *∆gpx* in the presence of uric acid (UA, panels A and B, respectively) and 4-aminobenzoic acid hydrazide (ABAH) (50 μM), apocynin (APO, 1 mM), or cytochalasin B (CYTB, 10 μg/mL) (panels C and D). Data were compared using one-way ANOVA followed by Bonferroni post-hoc and presented as mean ± standard error from six to seven independent triplicate experiments. *p < 0.05 compared with the WT or *∆gpx* groups; ^#^ *p *< 0.05 compared with the WT or *∆gpx* + neutrophils groups.

**Figure 3.**
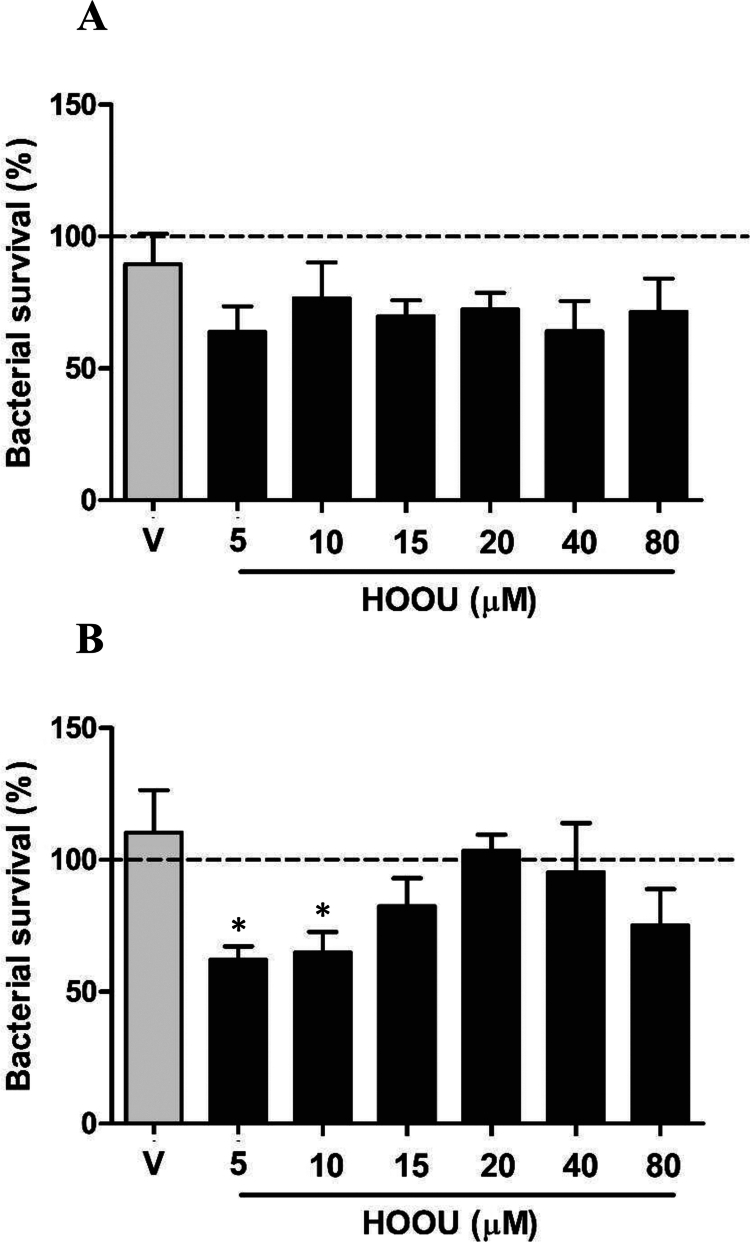
Survival of the wild-type (WT) PA14 (A) and *∆gpx* (B) strains to urate hydroperoxide (HOOU) *in vitro*. V: mobile phase (80 µL). Dashed lines represent the survival of the control. Data were compared using one-way ANOVA followed by Bonferroni post-hoc and presented as mean ± standard error of triplicates from three independent experiments. *p < 0.05 compared with vehicle control.

Approximately 41.5% ± 2.9% and 43.3% ± 2.1% of the WT and Δ*gpx* strains, respectively, survived incubation with human neutrophils. The addition of UA (200 and 400 µM), a substrate for MPO, significantly increased WT survival in a concentration-dependent manner (Figure 2A), possibly due to the diversion of MPO activity from the chlorinating cycle toward HOOU formation [58]. Conversely, 100 µM UA did not affect killing ability of neutrophils against Δ*gpx*, whereas a slight but statistically significant reduction in Δ*gpx* survival was observed with 200 µM UA (45.0% ± 2.2% vs. 35.7% ± 2.5%, *p* < 0.0001), a profile that was not found at the highest concentrations, 400 µM UA (Figure 2B). This result indicates that Δ*gpx* is sensitive enough that diversion on HOCl production by uric acid does not restore bacterial survival. Additionally, HOOU, a product from uric acid oxidation by neutrophils [58], can be toxic to Δ*gpx* but not to WT strain (Figure 3). Inhibition of MPO and flavoproteins by ABAH and apocynin, respectively, significantly increased WT survival (Figure 2C), suggesting that oxidative pathways are involved in WT death. However, these inhibitors did not affect Δ*gpx* survival showing that the strain may be sensitive enough to mild oxidative burst (Figure 2D). Survival of WT and Δ*gpx* was restored when neutrophils were pre-incubated with the phagocytosis inhibitor, cytochalasin B (10 µg/mL) (Figure 2C and D, respectively) [41].

Because uric acid induced a divergent effect on neutrophil killing against WT or Δ*gpx* strains, we incubated bacteria with purified HOOU at concentrations ranging from 5 to 80 µM. The WT strain exhibited no significant loss in viability at any of the tested HOOU concentrations after 30 min of incubation at 37 °C (Figure 3A). In contrast, the two lowest concentrations of HOOU led to death of approximately half of the Δ*gpx*. The survival of the mutant strain was restored at higher concentrations (Figure 3B). The mobile phase used in the purification of HOOU did not interfere with bacterial survival. These findings suggested that the GPx enzyme may be involved in the detoxification of organic peroxides (e.g. HOOU) formed during the oxidative burst.

**Figure 4.**
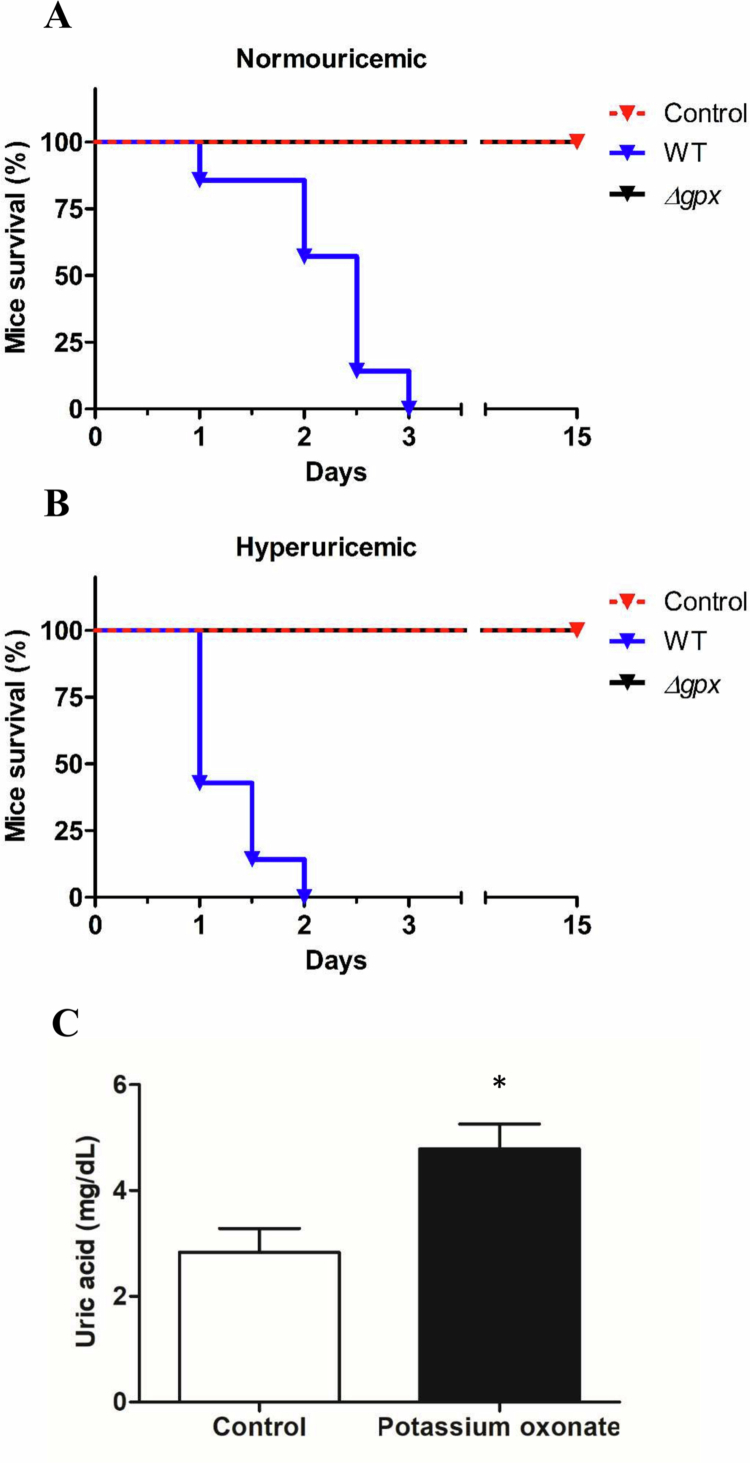
Survival of normouricemic (A) and hyperuricemic (B) C57BL/6 mice after intranasal instillation of sterile solution (non-infected), wild-type (WT), or Δ*gpx* (2.0 × 10^6^ colony-forming units per milliliter [CFU/mL]). Animals were monitored twice daily for survival and signs of distress for up to 15 days. Serum uric acid levels (C) were determined in mice after 15 days of continuous administration of potassium oxonate (300 mg/kg, i.p.) (*n* = 8 animals per group). Analyses were performed using the unpaired Student’s *t*-test. *p < 0.05 compared with controls (normouricemic group). The survival curves of *Δgpx*-infected (black line) and non-infected (control, red line) animals are fully overlapping in both normouricemic and hyperuricemic conditions.

The effects of GPx mutation on virulence and survival of PA14 in a mice model of lung infection were also assessed. As previously reported by our group, male mice infected with the WT strain succumbed within 3 days [15]. In contrast, no mortality was observed among ∆*gpx*-infected animals over the 15-day observation period, with survival curves fully overlapping those of the naïve (non-infected) group (Figure 4A). To explore the role of Δ*gpx* against different oxidants produced in PA14 infection, hyperuricemia was induced in mice via daily administration of potassium oxonate [42], followed by infection with the WT or *∆gpx*; the animals were monitored for 15 days. As expected [15], hyperuricemia accelerated mortality in mice exposed to the WT strain (Figure 4B); however, no effects were observed in *∆gpx*-infected mice as they remained alive and apparently healthy during the observation period (Figure 4B). After necropsy, no morphological alterations were observed in the organs of the group infected with *∆gpx* compared with controls (data not shown). After the experiment, blood samples were collected to measure serum UA levels, which were nearly twice as high in potassium oxonate-treated mice compared with the control group (Figure 4C).

**Figure 5.**
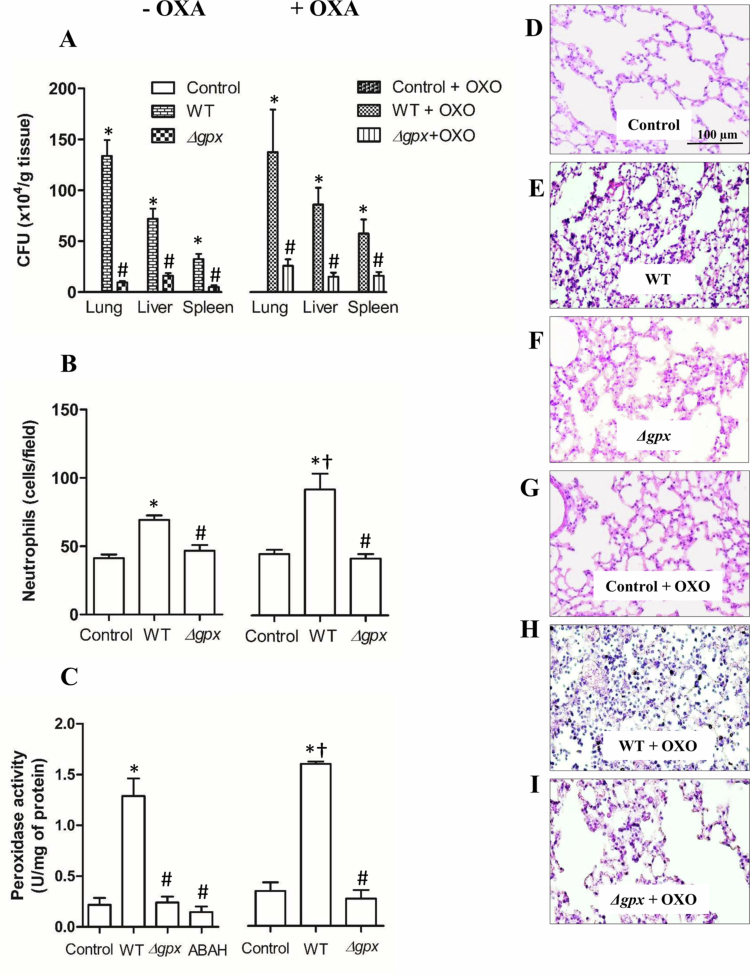
Bacterial load and neutrophil counts in *∆gpx*-infected mice. C57BL/6 mice were intranasally infected with 2.0 × 10⁶ bacteria (30 µL) of wild-type (WT) or *∆gpx* strains. Hyperuricemia was induced by potassium oxonate administration (OXO, 300 mg/kg, i.p.). Animals were euthanized after 22 h of infection, and tissues were collected via median laparotomy. The lung, spleen, and liver were homogenized in PBS-glucose, diluted, and plated onto Müeller-Hinton agar before colony-forming unit (CFU) counting (A). Neutrophil infiltration in the lung parenchyma was quantified (B), and peroxidase activity was measured by monitoring the TMB oxidation (C). 4-Aminobenzoic acid hydrazide (ABAH) (100 µM) was added to the WT homogenates as a control for myeloperoxidase-specific oxidation. Representative hematoxylin and eosin (H&E)–stained photomicrographs of lung sections from normouricemic animals (control [D], WT [E], and *Δgpx* [F]) and hyperuricemic animals (control [G], WT [H], and *Δgpx* [I]), showing basophilic nuclei (bluish-purple) and eosinophilic cytoplasm (pink). Neutrophils were counted in 20 random fields at 20× magnification. Data analyzed using one-way ANOVA followed by Bonferroni presented as mean ± standard error (*n* = 8 animals per group). *p < 0.05 compared to the respective control group; ^#^ *p *< 0.05 compared to the respective WT group; and ^†^ *p *< 0.05 compared to WT–OXO with WT + OXO.

A new cohort of normouricemic and hyperuricemic animals was intranasally infected with WT and Δ*gpx*. After 22 h, the lungs, liver, spleen, and bronchoalveolar lavage fluid were collected. Bacterial loads were several times higher in tissues from WT-infected mice than those infected with Δ*gpx*, regardless of hyperuricemia, suggesting a greater ability of the host to resolve the infection and/or reduce the *in vivo* survival of the mutant strain, despite the inflammation associated with hyperuricemia (Figure 5A).

Histological analysis of the lung parenchyma revealed that WT-infected animals exhibited a significantly greater number of neutrophils compared with *Δgpx*-infected and control mice under both normouricemic and hyperuricemic conditions. Neutrophil infiltration was significantly higher in hyperuricemic WT-infected mice than in normouricemic WT-infected animals (Figure 5B, 5D-I). In agreement with these findings, WT infection was associated with higher lung peroxidase activity compared with *Δgpx* infection in both normouricemic and hyperuricemic mice. The *Δgpx*-infected animals displayed a marked reduction in peroxidase activity, irrespective of uricemic status. Notably, hyperuricemic WT-infected mice exhibited significantly higher pulmonary MPO activity than normouricemic WT-infected animals (Figure 5C). To confirm that TMB oxidation was caused by MPO, ABAH (100 µM) was incubated with the homogenate of WT-infected normouricemic animals, and peroxidase activity was reduced to basal levels (Figure 5C).

**Figure 6.**
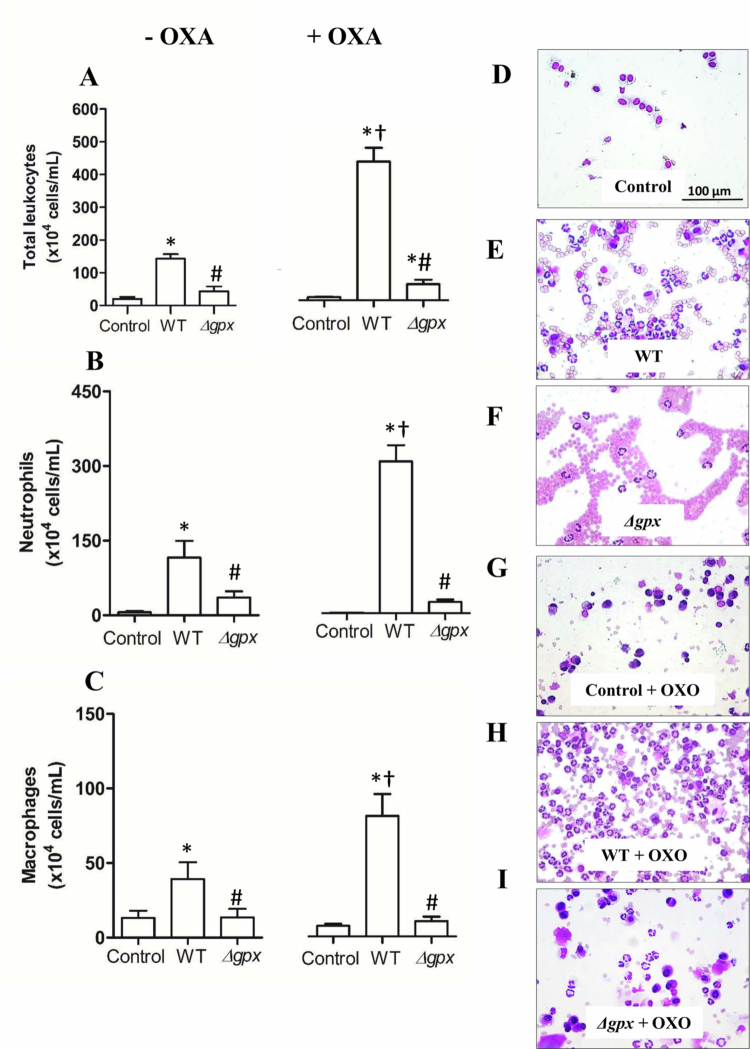
Quantification of total leukocytes (A), neutrophils (B), and macrophages (C) in bronchoalveolar lavage (BAL) fluid 22 h after intranasal inoculation with the wild-type (WT) and *∆gpx* (2.0 × 10^6^ colony-forming unit [CFU]). Hyperuricemia was induced by potassium oxonate administration (OXO, 300 mg/kg, i.p.). Representative photomicrographs of BAL fluid cell counts from normouricemic C57BL/6 mice stained with the Rapid Panoptic Kit (control [D], WT strain [E], and *Δgpx* mutant [F]) and hyperuricemic mice (control [G], WT strain [H], and *Δgpx* mutant [I]), showing dark blue–purple nuclei and cytoplasm ranging from light blue to pinkish tones. Data analyzed using one-way ANOVA followed Bonferroni post-hoc and expressed as mean ± standard error (*n* = 8 animals per group). *p < 0.05 compared to the respective control group; ^#^ *p *< 0.05 compared to the respective WT group; and ^†^ *p *< 0.05 compared to WT–OXO with WT + OXO.

**Figure 7.**
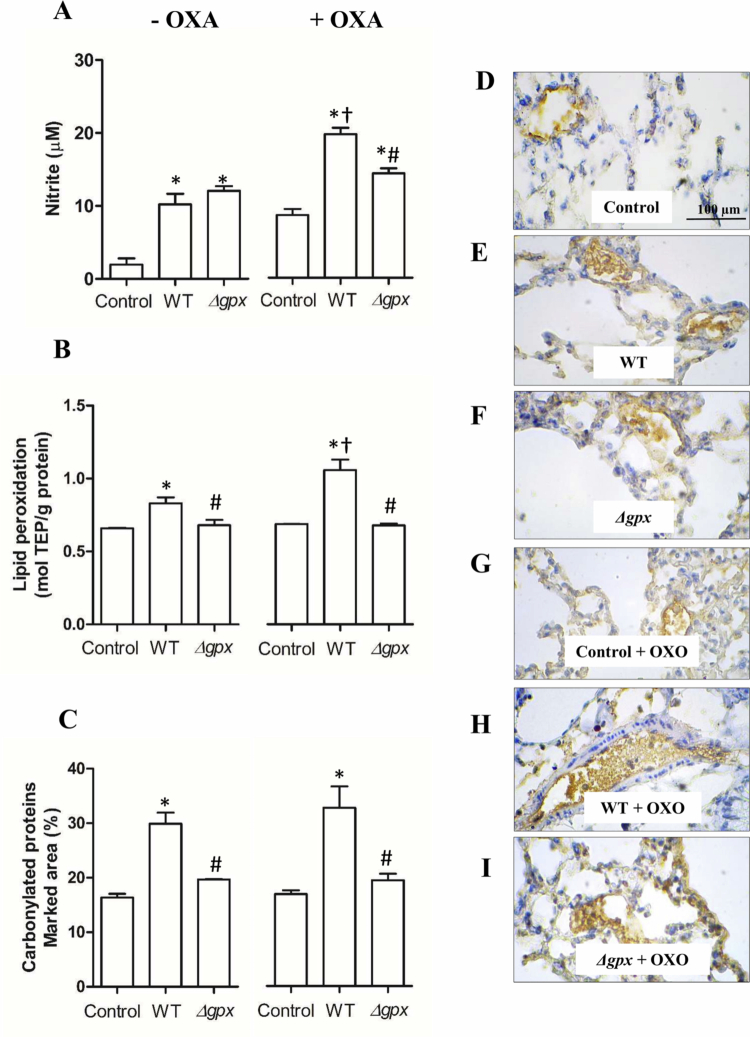
Nitrite concentration in bronchoalveolar lavage (BAL) (A), thiobarbituric acid reactive products (TBARs) (B), and carbonylated proteins (C). Hyperuricemia was induced by potassium oxonate administration (OXO, 300 mg/kg, i.p.). Representative photomicrographs of marked area for carbonylated (control [D], wild-type [WT] [E], and *∆gpx* [F]) and hyperuricemic animals (control [G], WT [H], and *∆gpx* [I]) evidenced by brownish staining. Data analyzed using one-way ANOVA followed by Bonferroni post-hoc and presented as mean ± standard error (eight animals in each group). *p < 0.05 compared to the respective control group; ^#^ *p *< 0.05 compared to the respective WT group; and ^†^ *p *< 0.05 compared to WT–OXO with WT + OXO.

**Figure 8.**
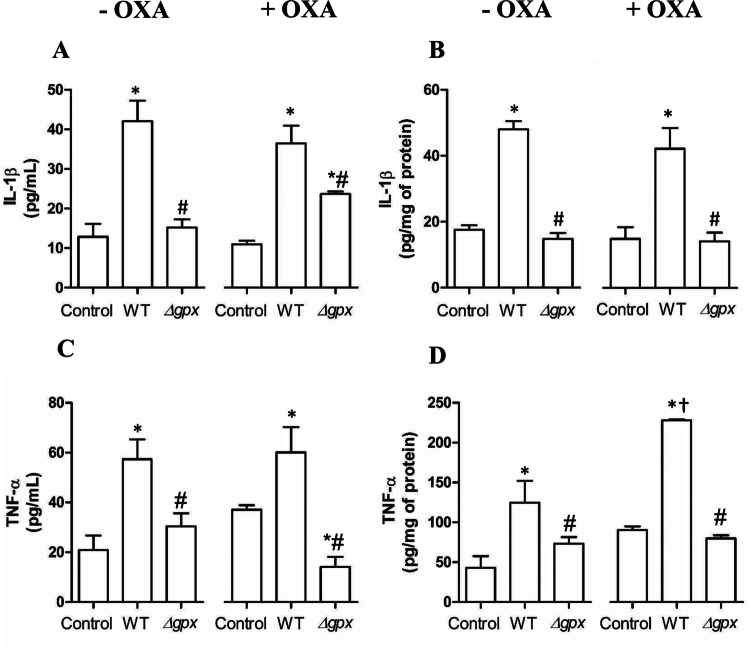
Cytokine levels in serum (A and C) and lung tissue (B and D). C57BL/6 mice were intranasally infected with 2.0 × 10^6^ bacteria from wild-type (WT) and *∆gpx*. Hyperuricemia was induced by potassium oxonate administration (OXO, 300 mg/kg, i.p.). After 22 h of infection, the animals were euthanized for cytokine measurement. Data analyzed using one-way ANOVA followed by the Bonferroni post-hoc. *p < 0.05 compared to the respective control group; ^#^ *p *< 0.05 compared to the respective WT group; and ^†^ *p *< 0.05 compared to WT–OXO with WT + OXO.

Lung infection with the WT strain increased the influx of total leukocytes (primarily neutrophils and macrophages) into the BAL (Figure 6). However, when mice were infected with the *∆gpx* strain, cell counts were comparable to those observed in the control group. Notably, hyperuricemic WT-infected mice exhibited a significantly higher number of total leukocytes in the BAL than normouricemic WT-infected animals. Consistent with the enhanced leukocyte influx into the BAL, nitrite and lipid peroxidation levels were significantly increased in hyperuricemic WT-infected mice compared with normouricemic WT-infected animals. Notably, nitrite levels were significantly reduced only in hyperuricemic *Δgpx*-infected mice. (Figure 7A and B). Regarding protein carbonylation, levels were significantly higher in WT-infected mice than in *Δgpx*-infected animals, regardless of their hyperuricemic state, as demonstrated by quantitative analysis (Figure 7C) and representative microscopy images (Figure 7D-I). Moreover, TNF-α and IL-1β levels were higher in WT-infected normouricemic and hyperuricemic mice than in *Δgpx*-infected animals in both serum (Figure 8A and C) and lung (Figure 8B and D). Notably, TNF-α levels in lung were significantly higher in hyperuricemic WT-infected mice compared with normouricemic WT-infected animals (Figure 8D). These findings suggest that the Δ*gpx* strain exhibits reduced virulence compared to the WT, regardless of the inflammation induced by hyperuricemia. Moreover, the exacerbated inflammatory and oxidative responses consistently observed in hyperuricemic WT-infected mice may help explain the increased mortality of WT-infected hyperuricemic animals in this study (Figure 4) and previously reported by our group [15].

## Discussion

This study was the first to demonstrate the involvement of GPx in the virulence of *P. aeruginosa* strain PA14. GPx enzymes are found in several bacteria (e.g. *Streptococcus pyogenes, Neisseria meningitides, Escherichia coli, Listeria monocytogenes*) [19,59] and play a crucial role in antioxidant defense, detoxification, and survival of the *P. aeruginosa* in the presence of various oxidants, particularly organic peroxides. Under physiological conditions, intracellular pro-oxidant agents and antioxidants maintain a dynamic balance; however, during infection, the host immune system is activated to control and eliminate the pathogen via oxidative and non-oxidative mechanisms [60].

Our results demonstrated that the GPx1 (PA14_27520) from PA14 contributed to bacterial survival, following exposure to activated human neutrophils and HOCl, H₂O₂, *t*-BOOH, or HOOU under *in vitro* conditions. H_2_O_2_ serves as a substrate of MPO to form HOCl from chloride ions during the chlorination cycle [61]. HOCl is the most potent oxidant generated in the inflammatory microenvironment, acting as a microbicidal agent capable of altering bacterial DNA [62], inhibiting its synthesis [63], and reacting with proteins and lipids [64]. Although GPx may contribute to resistance against these oxidants during neutrophil–bacteria interactions, its precise contribution to detoxification *in vivo* remains indirect. A previous study [16] demonstrated that the overexpression of GPx in *P. aeruginosa* increased the resistance of bacteria with mutations in the *katA* and *ohr* genes to H_2_O_2_ and organic peroxides. Another study involving *L. monocytogenes* demonstrated that GPx deletion increased bacterial tolerance to copper and iron, without affecting resistance to H₂O₂ [19], suggesting that the function of GPx may vary among bacterial species. In contrast to these observations, our findings indicate that GPx plays an important role in protecting PA14 against biologically relevant peroxides, particularly organic hydroperoxides, under conditions of oxidative stress.

UA is the end product of purine metabolism and acts as an important endogenous antioxidant. This antioxidant is present in biological fluids, primarily as monoanion urate (pKa = 5.4) and concentration between 50 and 400 µM in humans [20,21]. Urate is a physiological substrate for MPO, resulting in urate free radical, which subsequently reacts with O_2_•− to generate HOOU [22,58]. Furthermore, UA has a pro-inflammatory activity partially due to the activation of the NLRP3 inflammasome complex and produces IL-18 and IL-1β. The latter cytokine is possibly the main trigger of systemic inflammation and promotes extensive neutrophil infiltration and tissue remodeling [65]. In fact, UA has been described to contribute to the pathogenesis of several conditions, including hypertension, atherosclerosis, diabetes, and sepsis [20,66].

Our findings indicated that exposure to UA increased the survival of the WT strain PA14 in the presence of human neutrophils, possibly due to the reduction of MPO chlorinating activity. As a MPO substrate, UA competes with Cl^−^ and decreases HOCl synthesis, resulting in reduced bactericidal activity [23]. Additionally, UA may directly react with HOCl and produce stable, low-redox-potential products [21]. Although UA reduces MPO chlorinating activity [22], the formation of HOOU may be related to the lower survival of the *∆gpx* strain when neutrophils were incubated with 200 µΜ UA. Our results were consistent with previous findings [15] and indicated that low concentrations of HOOU (up to 10 µM) caused death of *∆gpx in vitro*, whereas the WT bacteria remained unaffected even at 80 μM. Thus, GPx contributes to bacterial resistance against HOOU and related oxidants during neutrophil-mediated oxidative stress, particularly in cellular and *in vitro* contexts.

To determine whether bacterial death resulted from oxidative or non-oxidative mechanisms, neutrophils were incubated with ABAH [37,38], apocynin [39,40], or cytochalasin B [41]. These findings indicate that GPx deficiency broadly compromises bacterial fitness during neutrophil interactions, rather than selectively increasing sensitivity to a single oxidative pathway. Other studies also showed that cytochalasin B reduced the death of *Staphylococcus aureus* 209P and *Streptococcus pyogenes* D58 by neutrophils [67] and the phagocytosis and killing of *S. aureus* 502 A and *E. coli* K12, without affecting bacterial opsonization [68].

*P. aeruginosa* is the major pathogen associated with acute pneumonia due to its biochemical versatility and widespread presence in clinical settings. In murine models, infection by this bacterium can progress to sepsis if not eliminated by components of the host immune response [69]. In our model of acute pneumonia using hyperuricemic mice, higher bacterial loads of the WT strain PA14 were collected in the lungs, spleen, and liver compared with normouricemic mice. Although the GPx mutant strain was also detected in the analyzed tissues, its bacterial load was significantly lower. This reduction in CFU could not be attributable to differences in growth rates, as the *Δgpx* and WT strains exhibited similar *in vitro* growth patterns. Accordingly, the reduced inflammation observed during *Δgpx* infection is most likely a consequence of decreased bacterial burden and impaired persistence in lung tissue, rather than a direct role of GPx in modulating the host immune response. A similar phenotype has been reported in other bacterial pathogens; for example, in a murine model of *Streptococcus pyogenes* infection, deletion of GPx resulted in a significantly lower number of bacteria recovered from infected tissues [59].

*P. aeruginosa* also promotes robust neutrophil recruitment to the infected respiratory tract. Within the lungs, this pathogen disrupts airway homeostasis, induces epithelial damage, and impairs immune responses [68]. In the BAL analysis, infection with the WT strain induced an increased influx of total leukocytes (mainly neutrophils and macrophages) into the bronchoalveolar fluid of both normouricemic and hyperuricemic animals, whereas leukocyte counts were reduced in mice infected with the *Δgpx* mutant in both conditions. Also, nitrite levels in the BAL peaked in hyperuricemic WT-infected mice, whereas a significant reduction was observed only in hyperuricemic animals infected with the *Δgpx* strain. MPO activity increased in infected animals under both normouricemic and hyperuricemic conditions, reaching even higher levels in hyperuricemic WT-infected mice. In contrast, MPO activity was reduced in *Δgpx*-infected animals in both conditions. This reduction in nitrite levels, together with the lower MPO activity observed in *Δgpx*-infected animals, indicates a faster resolution of the infection, consistent with the lower bacterial burden of this mutant. Notably, hyperuricemia further amplified oxidative and inflammatory responses during WT infection, and the magnitude of oxidative damage and inflammatory markers correlated with bacterial burden rather than differences in leukocyte recruitment *per se*. Lipid peroxidation resulted in pronounced membrane damage, as evidenced by elevated thiobarbituric acid reactive products, increased inflammatory cytokine levels, anti-DNP staining in the lung parenchyma, and higher levels of carbonylated proteins. These alterations were most evident in hyperuricemic WT-infected mice. Consistent with this tissue injury, *P*. aeruginosa infection was associated with disruption of lung architecture, likely driven by sustained neutrophilic inflammation and mucus accumulation [6,69]. The levels of TNF-α, but not IL-1β, were elevated in hyperuricemic animals infected with the WT strain, suggesting that hyperuricemia preferentially amplifies TNF-driven inflammatory pathways during WT infection [70]. The absence of a parallel increase in IL-1β may reflect its tighter regulation and dependence on inflammasome activation, which is not necessarily enhanced by soluble hyperuricemia in acute pulmonary infection. Importantly, although uric acid has been widely described as an activator of IL-1β through NLRP3 inflammasome signaling, this effect appears to be context-dependent and may not predominate during acute pulmonary infection with *P. aeruginosa* [71–74]. Taken together, these inflammatory profiles indicate that the exacerbated host response observed during WT infection — particularly under hyperuricemic conditions — is primarily driven by increased bacterial persistence rather than by direct modulation of host immune pathways. Accordingly, GPx deletion increases bacterial susceptibility to the host innate immune response, thereby limiting tissue damage and inflammation without requiring direct immunomodulatory effects [15,51].

Rocha et al. [15] demonstrated that normouricemic mice infected with *ΔahpC1* survived for 15 days, whereas all animals intranasally infected with WT or *ΔahpC1 attB:ahpC1* succumbed before the third day of infection. Under hyperuricemic conditions, all WT-infected animals died within 24 h, whereas approximately 40% of mice infected with the *ΔahpC1* strain succumbed within 48 h and all animals died after 3.5 days. Our results were consistent with those findings since normouricemic mice infected with WT succumbed within 3 days, whereas those infected with *Δgpx* survived for 15 days. Notably, WT-infected hyperuricemic mice succumbed even earlier than normouricemic WT-infected animals, possibly due to the exacerbated inflammatory and oxidative milieu observed in this group, rather than reduced chlorinating activity of neutrophils alone. On the other hand, all animals infected with *Δgpx* remained alive for 15 days despite the hyperuricemic state, highlighting the importance of GPx for PA14 fitness and virulence *in vivo*. A previous study using *S. pyogenes* also demonstrated that mice infected with the WT strain succumbed on the fourth day after infection, whereas the group infected with the mutant strain lacking the GPx enzyme survived for at least 9 days [59]. The apparent differences between *Δgpx* and *ΔahpC1* PA14 phenotypes raise the possibility of distinct temporal roles of bacterial peroxidases during infection. AhpC1 may be predominantly required to cope with exacerbated oxidative stress once infection is established, whereas GPx may play a more critical role during the early stages of infection to sustain bacterial survival and persistence. In this context, loss of GPx is associated with complete attenuation of virulence regardless of uric acid status, while loss of AhpC leads to a conditional attenuation that becomes evident only under hyperuricemic conditions. These hypotheses, however, warrant further experimental validation.

This study identifies a previously unrecognized role for bacterial glutathione peroxidase in shaping the outcome of *P. aeruginosa* infection. The reduced inflammatory response observed in animals infected with the *Δgpx* mutant occurred in parallel with markedly lower bacterial burdens, supporting the interpretation that impaired bacterial fitness and persistence are the primary drivers of this phenotype rather than direct modulation of host immune activation by GPx. Within this framework, the increased susceptibility of the *Δgpx* strain to oxidative killing reflects an inability to withstand host-derived oxidative stress during infection, particularly under conditions of exacerbated inflammation.

Although the present data clearly demonstrate a critical role for GPx in bacterial survival and virulence, the precise molecular mechanisms by which this enzyme confers resistance to specific oxidants *in vivo* remain to be fully elucidated. Future studies integrating biochemical, genetic, and structural approaches will be important to further dissect GPx-dependent redox pathways and their contribution to bacterial fitness during infection.

An additional consideration raised by these findings concerns the potential translational relevance of the specific glutathione peroxidase studied here (*gpx*, PA14_27520) as a therapeutic target. GPx-like enzymes constitute a heterogeneous family with substantial sequence and structural divergence across biological kingdoms, and *P. aeruginosa* encodes multiple peroxidases with overlapping yet non-redundant functions. Comparative *in silico* analyses using BLAST (Supplementary file) revealed limited overall sequence coverage between PA14 GPx and human GPx isoforms, with similarity restricted to short and discontinuous regions, indicating low global homology. These findings suggest that structural divergence may permit selective targeting of the bacterial enzyme; however, they do not exclude the possibility of partial off-target interactions with host antioxidant systems or members of the commensal microbiota. Notably, no inhibitors have been specifically developed against bacterial GPx to date, and compounds described as GPx inhibitors have primarily been characterized in mammalian systems without selectivity profiling against bacterial homologs. Comprehensive structure-based analyses and functional validation studies would therefore be required to evaluate the feasibility and safety of targeting bacterial GPx. Such investigations were beyond the scope of the present study.

In conclusion, this study demonstrates a crucial role for glutathione peroxidase in the survival and virulence of *P. aeruginosa* PA14 under inflammatory conditions. GPx contributes to bacterial resistance against host-derived oxidative stress during interactions with neutrophils and is required for efficient bacterial fitness and persistence *in vivo*. The reduced inflammation and tissue damage observed during *Δgpx* infection are best explained by the markedly lower bacterial burden, rather than by direct modulation of host immune responses. Collectively, these findings highlight the importance of bacterial redox defenses in shaping infection outcomes and support GPx as a relevant determinant of *P. aeruginosa* pathogenicity, reinforcing redox regulation as a promising framework for anti-virulence strategies against multidrug-resistant pathogens.

## Supplementary Material

Author Checklist_RR updated.pdf Author Checklist_RR updated.pdf

Supplementary material.docx Supplementary material.docx

## Data Availability

Data and materials are available from the corresponding author upon reasonable request.
