# Supplementary figures and images for: Redox protection by bacterial glutathione peroxidase drives virulence in Pseudomonas aeruginosa
Source: Redox Rep. 2026 May 22;31(1):2676357. doi: 10.1080/13510002.2026.2676357 (PMC13202656; doi:10.1080/13510002.2026.2676357)

**Figure S1**


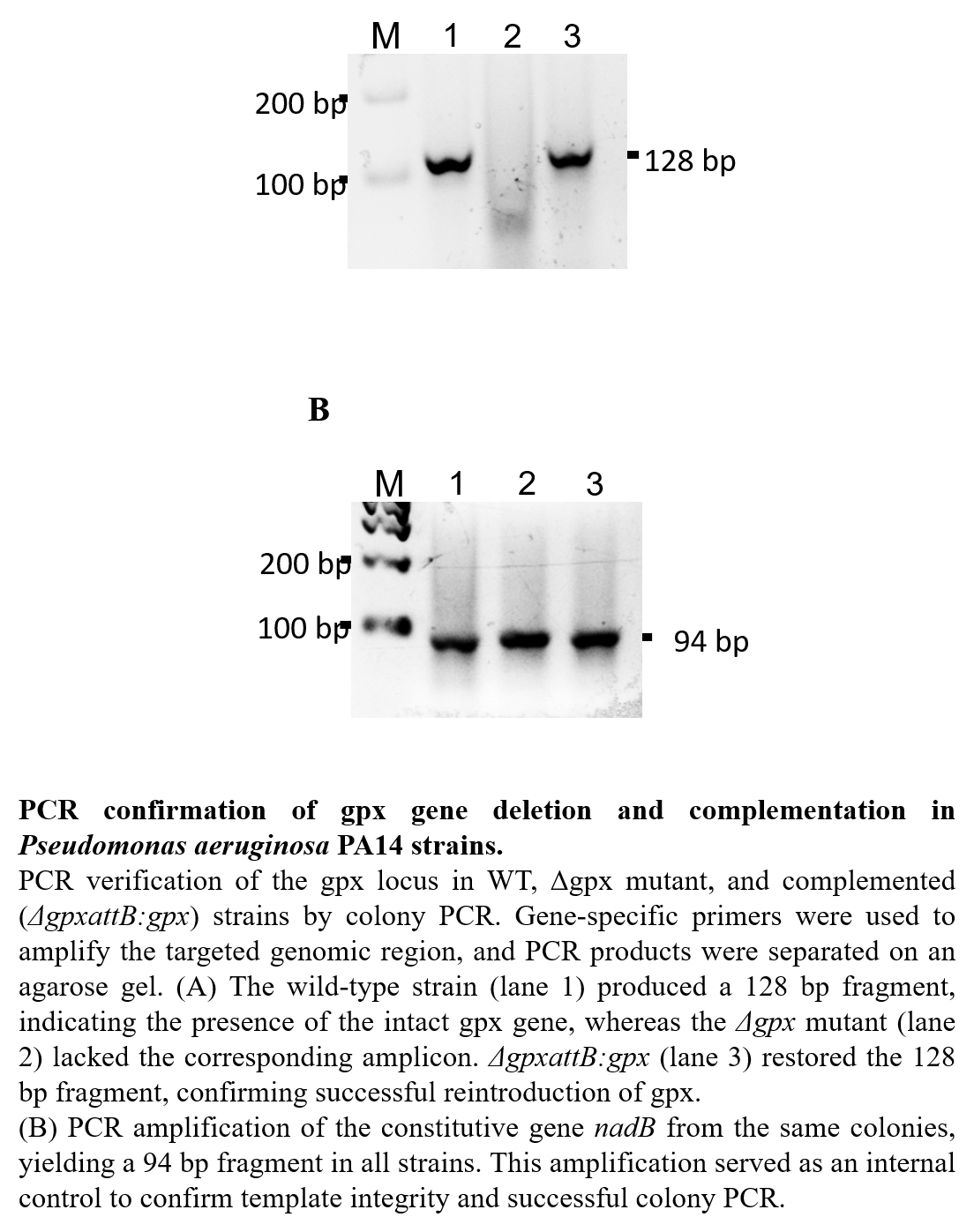


**Figure S2**

**
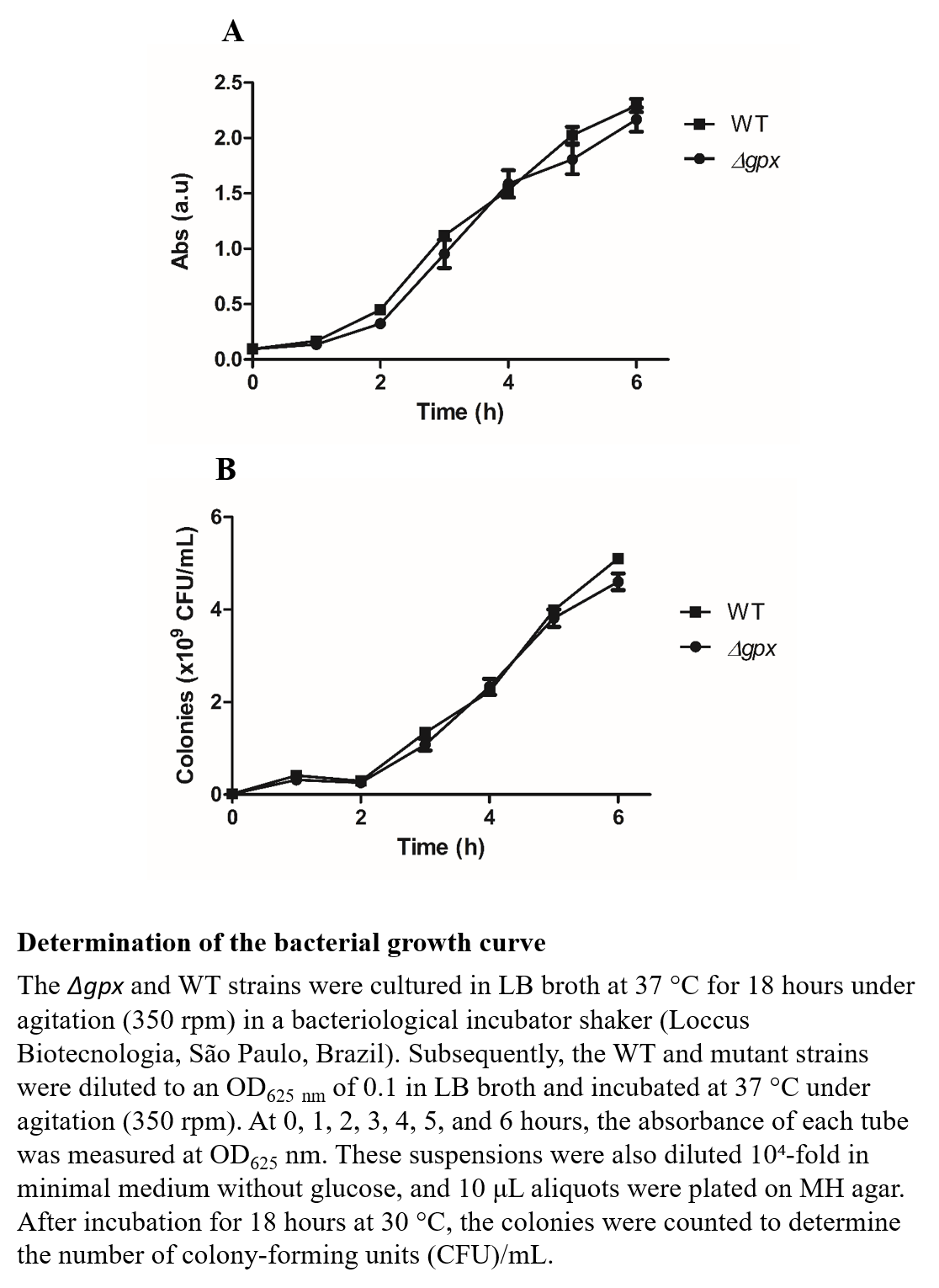
**

Supplement: Supplementary material.docx [file YRER_A_2676357_SM0334.docx]
